# Role of Dendritic Cells in the Induction of Lymphocyte Tolerance

**DOI:** 10.3389/fimmu.2015.00535

**Published:** 2015-10-20

**Authors:** Fabiola Osorio, Camila Fuentes, Mercedes N. López, Flavio Salazar-Onfray, Fermín E. González

**Affiliations:** ^1^Millennium Institute on Immunology and Immunotherapy, Faculty of Medicine, University of Chile, Santiago, Chile; ^2^Disciplinary Program of Immunology, Institute of Biomedical Sciences, Faculty of Medicine, University of Chile, Santiago, Chile; ^3^Cell Therapy Laboratory, Blood Bank Service, University of Chile Clinical Hospital, Santiago, Chile; ^4^Laboratory of Experimental Immunology and Cancer, Faculty of Dentistry, University of Chile, Santiago, Chile

**Keywords:** dendritic cells, DC-based immunotherapy, tolerance, Foxp3, regulatory T cells

## Abstract

The ability of dendritic cells (DCs) to trigger tolerance or immunity is dictated by the context in which an antigen is encountered. A large body of evidence indicates that antigen presentation by steady-state DCs induces peripheral tolerance through mechanisms such as the secretion of soluble factors, the clonal deletion of autoreactive T cells, and feedback control of regulatory T cells. Moreover, recent understandings on the function of DC lineages and the advent of murine models of DC depletion have highlighted the contribution of DCs to lymphocyte tolerance. Importantly, these findings are now being applied to human research in the contexts of autoimmune diseases, allergies, and transplant rejection. Indeed, DC-based immunotherapy research has made important progress in the area of human health, particularly in regards to cancer. A better understanding of several DC-related aspects including the features of DC lineages, milieu composition, specific expression of surface molecules, the control of signaling responses, and the identification of competent stimuli able to trigger and sustain a tolerogenic outcome will contribute to the success of DC-based immunotherapy in the area of lymphocyte tolerance. This review will discuss the latest advances in the biology of DC subtypes related to the induction of regulatory T cells, in addition to presenting current *ex vivo* protocols for tolerogenic DC production. Particular attention will be given to the molecules and signals relevant for achieving an adequate tolerogenic response for the treatment of human pathologies.

## Introduction

Dendritic cells (DCs) are key controllers of innate and adaptive immunity and central regulators of immune tolerance (1). These functions are mainly due to the capacities of DCs to capture and process antigens and respond to pathogen- and self-derived danger signals in peripheral tissues. Additionally, migratory abilities allow competent DCs to mobilize into draining secondary lymphoid organs where they engage, in the presence of specific cytokines, antigen-specific naïve CD4^+^ and CD8^+^ T cells, thus triggering the activation, proliferation, and migration of these cells to peripheral tissues (2, 3).

It has become increasingly clear in recent years that DCs are a heterogeneous population of leukocytes. Various distinct DC subtypes have been identified, including plasmacytoid DCs (pDCs), conventional DCs (cDCs), and inflammatory DCs (iDCs), the last of which display most of the archetypical features of DCs and originate from circulating monocytes during inflammation (4–7). The cDC subtype can be divided further, with mice presenting the CD8α^+^/CD103^+^ and CD11b^+^ subsets (8), and the human counterparts that express the markers BDCA-3 and BDCA-1, respectively (5). Even though DC subtypes share common features, such as activation in response to pathogen recognition, each DC population has specialized functions (4). For example, pDCs produce type I interferon for viral immunity (9, 10), and iDCs display antimicrobial functions by producing the tumor necrosis factor (TNF) and the inducible nitric oxide synthase during infections (5, 11). In the case of cDCs, these cells are highly efficient at coupling innate and adaptive immunity (8). Moreover, the CD8α^+^/CD103^+^ cDC subset excels in the cross-presentation of antigens to cytotoxic CD8^+^ T cells, while CD11b^+^ cDCs efficiently prime naïve CD4^+^ T cells (12).

Despite the identification of the DC lineages, it is currently unclear if there is a DC subtype with dedicated tolerogenic functions. Indeed, rather than a specialized population, evidence indicates that most DC subtypes can promote lymphocyte tolerance through T cell deletion, T cell anergy induction, and regulatory T cells (Tregs) induction (13). Furthermore, the tolerogenic features of DCs can be influenced by the microenvironment, expression of specific receptors that favor antigen presentation in subimmunogenic conditions, activation of specific intracellular signaling responses, and by pharmacological modulation, among other factors. A better understanding of these mechanisms will support the development of DC-based therapies that induce specific Tregs, which can be applied in the treatment of immune system pathologies that display over-activation against self-antigens as well as for the treatment of transplant rejection.

For the purpose of this review, we will provide an overview of the function of DCs in the establishment of lymphocyte tolerance, with focus on Tregs induction. We will also provide perspectives on the mechanisms that could be exploited to induce tolerogenic DCs (TolDCs) in the context of human immune diseases.

## Contribution of DCs to the Induction of Natural and Peripheral Tregs

Dendritic cells have long been candidates for immunotherapy (14). In mice, these cells can be used to efficiently expand antigen-specific or polyclonal Tregs in autoimmune, transplant, and graft-versus-host diseases, among other immunological disorders (15–18). With the advent of intravital microscopy, the *in vivo* interactions between DCs and Tregs in lymphoid organs have been visualized (19). Related to this, some studies have found that endogenous Treg-DC interactions in the lymph node last longer than those established between DCs and conventional T cells in steady-state or during inflammation (20), thus highlighting the relevance of Treg and DC contacts in physiological conditions.

The establishment of central tolerance in the thymus predominantly depends on medullary thymic epithelial cells, which express the autoimmune regulator AIRE, a transcription factor required for the presentation of tissue-specific antigens to thymocytes (21, 22). In this context, the role of DCs in central tolerance appears limited, as demonstrated by studies showing that the thymic T cell compartment is unimpaired in mice displaying constitutive ablation of DCs (23, 24). However and despite these findings, it has been suggested that DCs could play a non-redundant role in the induction of natural Tregs (nTregs) (23, 25, 26). An advanced study of the T cell receptor repertoire in thymocytes showed that bone marrow-derived antigen-presenting cells (APCs), which include DCs, substantially contribute to the composition of the nTreg T cell receptor repertoire (26). Interestingly, CD8α^+^ cDCs can acquire and present autoimmune regulator-dependent antigens derived from medullary thymic epithelial cells to nTregs (26).

In addition to sustain nTreg selection, DCs can promote nTreg development in the thymus via the CD27–CD70 costimulation axis (27). Expression of CD70 on epithelial cells and DCs, particularly those of the CD8α^+^ subset, contributes to nTreg survival (27). Thus, there is growing evidence indicating that DCs could be actively involved in promoting thymic Treg development. Nevertheless, it remains to be discovered if the repertoire, frequency, and function of nTregs are dependent on a specialized DC subset.

In secondary lymphoid organs and in non-lymphoid tissues, the dynamics of DC and Treg populations are closely intertwined (28). This notion is derived from observations in Foxp3^DTR^ mice, in which the depletion of Tregs leads to a notable increase in DCs frequency (29). The mechanism by which Tregs exert control over DC expansion is not fully understood, but it does depend on the cytokine Flt3L, a crucial regulator of DC commitment (8, 30). In fact, *in vivo* Flt3L treatment results in more DCs and a concomitant increase in the number of inducible Tregs (iTregs) (31). In contrast, *Flt3l*^−/−^ mice show a 10-fold reduction in cDCs together with lower Treg cell counts (28). Even though evidence suggests that Flt3L leads to Treg expansion via a DC-dependent mechanism, this cytokine may also influence additional myeloid cell types, which in turn could impact Treg homeostasis independently of DCs. Nevertheless, data acquired from models of acute DC depletion, as triggered by administering a diphtheria toxin to CD11c-DTR mice, also demonstrated a two- or threefold reduction of iTregs (28, 32). These findings indicate that DCs are active regulators of Treg numbers *in vivo*.

Also in peripheral tissues, the maintenance of Tregs is dependent on costimulation by DCs. Despite some reports indicating that the absence of CD80/86 molecules on DCs leads to higher expression of Foxp3 (33), additional work has revealed that interactions mediated by CD80 and CD86 on APCs with the CD28 receptor on T cells are key for maintaining iTregs (34–37). These studies have been recently revisited in experiments using CD11c DTA mice (38), which constitutively lack DCs (32). Findings demonstrate that the absence of CD80/86 in the CD11c^+^ compartment results in a reduction of peripheral Tregs (32). Thus, in addition to major histocompatibility complex (MHC)-II molecules (28), iTreg maintenance critically relies on the expression of costimulatory molecules by DCs (32).

Another mechanism through which DCs can maintain Treg functions is via expression of the programed cell death ligand-1 (PD-L1) molecule. Interaction between PD-L1 on DCs and PD-1 receptor on T cells promotes the expression of Foxp3^+^ Tregs, whereas blocking PD-L1 *in vivo* curtails Treg conversion during tumor challenge (39). Furthermore, the PDL1/2-PD1 interaction between DCs and Treg cells prevents autoimmunity of the central nervous system (40). In addition to the activation molecules, the migratory abilities of DCs may be relevant in promoting T cell tolerance. Indeed, migratory DCs have superior abilities than resident DCs to induce Treg development for the same antigen (41), which may be important to consider in the design of DC-based therapies.

Altogether, these findings indicate that DCs promote the induction of natural and iTregs via a variety of mechanisms that include the expression of multiple costimulatory and coinhibitory signaling pathways, the secretion of regulatory factors, migration, and the ability to present antigens. The advent of innovative models for selective ablation of cDCs using discriminating markers, such as the zinc finger transcription factor, zDC or the DC receptor, DNGR-1 (42, 43), will provide invaluable information toward addressing the role of DC lineages in the establishment of lymphocyte tolerance.

## Contribution of DC Subtypes to T Cell Tolerance

Current understanding dictates that antigen presentation in the absence of pathogen/danger detection leads to T cell tolerance of self or non-self antigens (4, 13). Nevertheless, continuing discoveries on the complexity and diversity of the DC network make it necessary to reassess current knowledge of DC subsets in the establishment of T cell tolerance. In particular, markers historically attributed to DCs, such as MHC-II and the integrin CD11c, do not actually discriminate among DC subpopulations and often overlap with additional members of the mononuclear phagocyte system, including monocytes and macrophages, especially under inflammatory conditions (44). Through the discovery of specific DC markers and a review of DC lineages based on ontogeny, researchers will be able to better understand the contribution of DC subtypes to Treg development in a variety of scenarios.

### CD8α^+^/CD103^+^ cDcs

Conventional DCs expressing the CD8α^+^ marker can be found in lymphoid organs while cDCs located in non-lymphoid tissues express the marker CD103^+^, with the exception of a subpopulation of DCs in the intestine (5, 7, 45, 46). The CD8α^+^/CD103^+^ subsets share a common origin and function and depend on the transcription factors Batf3, IRF8, and Id2 for development (6, 8, 44, 46–48). Given the ability of these subsets to cross-present antigens, CD8α^+^ and CD103^+^ DCs are traditionally associated with the cross-priming of pathogens- and tumor-derived antigens to CD8^+^ T cells (5). Moreover, in the absence of inflammation, CD8α^+^/CD103^+^ DCs can also regulate the induction of Tregs and mediate T cell tolerance (8, 21). Functionally, splenic CD8α^+^ DCs are superior at inducing Foxp3^+^ T cell differentiation than CD8α^−^ DC counterparts due to their ability to produce transforming growth factor beta (TGF-β) (39, 49). Furthermore, CD8α^+^ cells have demonstrated proficient abilities to induce iTregs in *in vivo* studies of antigen targeting via the expression of antibodies specific to endocytic receptors, such as the C-type lectins DEC205 and DNGR-1 (50, 51). These results indicate that CD8α^+^ DCs can be manipulated to induce iTreg differentiation *in vivo* by means of antigen delivery.

In tissues, CD8α^+^/CD103^+^ DCs related to the gut-associated lymphoid tissue (GALT) are critically involved in oral tolerance and in inducing the *de novo* differentiation of Foxp3^+^ T cells (52). However, the ability of CD8α^+^/CD103^+^ DCs to induce iTregs *in vivo* is not exclusive, as indicated by studies in *batf3*^−/−^ mice, which lack the CD8α^+^/CD103^+^ DC subsets but display normal frequencies of intestinal Tregs, indicating that additional APCs can compensate to safeguard Treg homeostasis in the intestine (45).

### CD11b^+^ cDC

Conventional DCs expressing CD11b^+^ are found across lymphoid organs and non-lymphoid tissues, but their function in tolerance has been challenging to address, partially since these cells express markers that are shared with macrophages and monocytes, such as CD11c, MHC-II, and CD11b (7). Nevertheless, in inflamed skin, dermal CD11b^+^ DCs display a marked ability to induce Foxp3^+^ T cells (53). Furthermore, the capacity of DCs to mediate Treg induction in the dermis is attributable to the CD11b^+^CD103^−^ subtype (54). These findings indicate that CD11b^+^ DCs can regulate the homeostasis of Tregs in the skin.

Nevertheless, and as noted for CD103^+^ DCs, the selective ablation of CD11b^+^ DCs does not alter Treg frequencies, indicating that these cells may be sufficient but not necessary for Treg cell homeostasis (55). The discovery of new surface molecules allowing for the distinction of *bona fide* CD11b^+^ DCs such as CD24, CD64, and MerTK will provide relevant information on this cell type in Treg induction.

### Plasmacytoid DCs

Plasmacytoid dendritic cells found in the bone marrow and lymphoid organs, which express the markers CD11c, B220, Siglec-H, and BST2, depend on the E2-2 transcription factor for development (7) and produce large amounts of type I interferon in response to viral recognition (7). Although the involvement of pDCs in antigen presentation to T cells is a matter of debate (56), MHC-II-restricted antigen presentation by pDCs promotes Foxp3^+^ T cell expansion that results in protection against experimental autoimmune encephalomyelitis (57). Furthermore, pDCs with dietary antigens can mediate oral tolerance via CD4^+^ and CD8^+^ T cells (58) and induce tolerance to vascularized cardiac allografts via the generation of Foxp3^+^ T cells (59). In humans, pDCs isolated from blood induce Foxp3^+^ T cells (60), which is attributed to the expression of the enzyme indoleamine 2,3-dioxygenase, PD-L1, and the inducible costimulator-ligand (10, 61, 62). In addition, pDCs residing in the human thymus drive nTreg development *ex vivo* (63), and stimuli such as cytosine-phosphate-guanine and the thymic stromal lymphopoietin have shown to promote this function (64). These findings indicate that human pDCs have the potential to be used as tools for Treg induction. In contrast to human counterparts, murine pDCs display limited abilities to induce Tregs (10). On the other hand, antigen targeting to the human BDCA-2 molecule in B6.BDCA2 transgenic mice results in antigen-specific tolerance and an increased Tregs/T effector ratio (65).

Altogether, current evidence indicates that pDCs strongly contribute to Treg homeostasis and could be attractive candidates for immunotherapy treatments against several conditions, such as transplant rejection, autoimmune diseases, and cancer (66).

### Inflammatory DCs

Inflammatory dendritic cells present during infection/inflammation, are derived from monocytes, and express the markers CD11c, MHC-II, CD11b, and CD64. iDCs release large amounts of inflammatory cytokines upon pathogen recognition, including TNF and the inducible nitric oxide synthase (7). Moreover, iDCs drive Th1- and Th17-mediated immunity, a feature also recorded in humans (67). Given that these cells are not present in steady-state conditions, their role in maintaining Treg homeostasis *in vivo* is not fully understood. Nevertheless, *in vitro* models of DC generation have been widely used. DCs stimulated by the granulocyte macrophage colony-stimulating factor (GM-CSF), plus or minus interleukin (IL)-4, induce large numbers of cells that resemble those originated under inflammatory contexts (8, 68), and these cells have been broadly used as tools to differentiate Tregs *ex vivo* (15, 69). However, recent findings indicate that GM-CSF-derived DCs are composed by a heterogeneous mixture of macrophages and DCs, which are indistinguishable through quantification of CD11c and MHC-II expression (70). It is also currently unclear which population is mediating Treg induction.

Thus, current data indicate that while DCs facilitate the induction of Tregs, there is no strict dependency on any DC lineage to sustain this function. Rather, it seems that several immune cell types, depending on the scenario, can contribute to the regulation of Treg cell homeostasis.

## Role of Exogenous and Endogenous Triggers of Tolerogenic DCs

The ability of DCs to favor Treg homeostasis and restrict T cell immunity can be fine-tuned by several extracellular and intracellular factors. These factors include microenvironmental features, the expression of surface molecules, and the selective activation of specific signaling pathways that promote Treg induction. In addition, TolDCs have been generated and studied in contexts of pharmacological modulation. The aspects that contribute to the programing of DCs in regards to triggering T cell tolerance are described below.

### Microenvironment

The homeostasis of Tregs can be specifically regulated depending on location, metabolism of dietary products, or exposure to microbes or tumors (71, 72). In the microenvironment of a tumor, cancer cells can produce suppressive factors that promote DCs to induce Tregs that can, in turn, contribute to tumor progression (71). For example, DCs isolated from tumor-bearing animals are programed by cancer cells to promote Treg proliferation via TGF-β production (73). Moreover, apoptotic cells resulting from physiological processes are potent inducers of tolerance via DCs (74). Interestingly, early traslocation of annexin A1 contributes an essential tolerogenic property to apoptotic cells, conferring their ability to suppress DC activation and cellular immunity (75). Annexin A1 on apoptotic cells critically interferes with TLR signaling pathways upstream of NFκB activation and, in turn, preventing antigen-specific T cell responses (75). In addition, clearance of dead cells by DCs, which requires dedicated receptors such as DEC205 (76), results in the induction of Tregs (77). In *in vivo* models, the apoptotic cargo is preferentially captured by the CD8α^+^ lineage of DCs, which are able to mediate tolerance to cell-associated antigens (78).

Another characteristic site for Treg presence is the gut (52, 71, 72, 79, 80). Here, GALT–CD103^+^ DCs specialize in the synthesis of retinoic acid (RA) and are the cell type predominantly responsible for Treg generation (52, 79). RA is a metabolite of vitamin A that regulates Treg induction by enhancing TGF-β-induced conversion of CD4^+^ T cells to Foxp3^+^ T cells (33, 81, 82). Specifically, RA sustains the differentiation of Foxp3^+^ T cells by acting *in cis* on naïve T cells or *in trans* by relieving the inhibition exerted by CD4^+^ memory T cells on iTreg development (33, 81, 82). CD103^+^ DCs isolated from the small intestine lamina propria can synthesize RA and secrete bioactive TGF-β at levels sufficient to trigger Treg differentiation (52, 71). Notably, the effect of RA in promoting Tregs is not restricted to the intestine. In the skin, RA production is attributed to dermal CD11b^+^ DCs, which trigger *de novo* iTreg differentiation with higher efficiency than CD103^+^ counterparts (54). Even though the tissue-associated signals that lead to RA production by distinct DC subsets in the gut and skin are unclear, these findings indicate that the ability to induce iTregs is not an attribute of a dedicated DC subtype and that the outcome of the immune response is dictated by the integration of multiple environmental cues.

In a physiological context, DCs may also contribute to the maintenance of Treg homeostasis through the induction of tolerance to commensal microbiota (83, 84). Although the predominating standpoint is that DCs activated with microbes via pattern recognition receptors (PRRs) provide the signals necessary to elicit T cell immunity, it has been also reported that Tregs can arise in non-sterile conditions. This notion is supported by studies in germ-free mice, which display reduced Tregs frequency (85, 86). In fact, certain intestinal bacteria, including *Clostridium* strains and the human commensal bacteria *Bacteroides fragilis*, have been found to promote Treg development (85, 87). These findings open new avenues for studying the role of DCs in Treg homeostasis in the context of microbe recognition, particularly against commensal and/or symbiotic microbiota. However, extensive studies must be carried out to reveal the mechanisms by which these particular microbes are able to induce Tregs and prevent T cell immunity or even Treg plasticity. Identification of these particular signatures would be greatly relevant to the advancement of DCs use in the treatment of diseases as there are additional microbial compounds, such as the fungal β-glucan curdlan, that trigger reprograming of Tregs into Th17 cells, which may result in immunity rather than tolerance depending on the conditions (88).

The *in vivo* identification of microbial-derived components that can trigger Tregs is an emerging field with many prospects related to clinical immunology. Along these lines, it remains to be determined whether the effects of commensal microbiota are indeed mediated by DC subtypes.

## Surface Molecules and Activation of Signaling Responses

Dendritic cell function can be modulated by the expression of surface molecules and activation of signaling cascades responsible for promoting Treg cells. In addition to the endocytic receptors such as DEC205 that can be exploited for Treg induction, certain surface molecules can confer tolerogenic properties to DCs. On one hand, expression of the TGF-β activating integrin αvβ8 by DCs is required for efficient Treg induction (89, 90). In fact, mice deficient in αvβ8 in DCs have decreased abilities to generate iTregs. This limitation causes autoimmunity and colitis in mice (89, 90), indicating that TGF-β processing by DCs is a key feature for maintaining Treg homeostasis.

**Figure 1:**
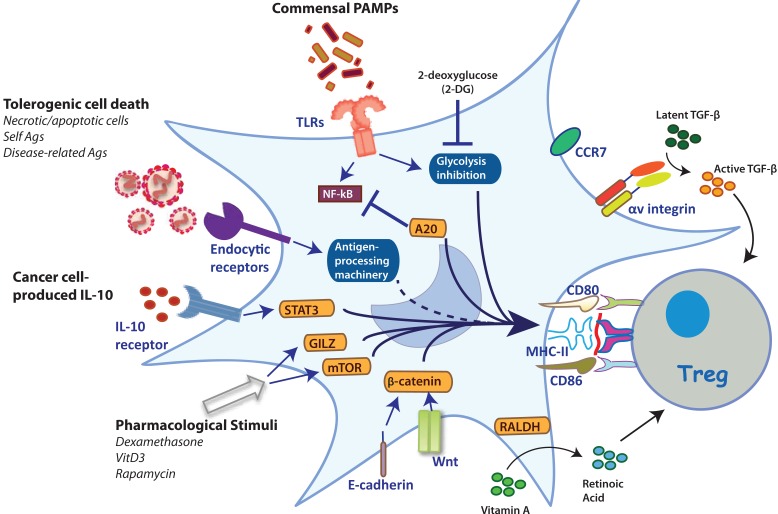
**Stimuli and mechanisms that induce the generation of TolDCs**. Several stimuli can trigger different intracellular mechanisms that reprogram TolDCs to induce Tregs. TLR2 triggering results in Treg induction (91). These include activation via TLR2, expression of αv integrin for activation of TGF-β, and expression of endocytic receptors responsible to divert the antigenic cargo for presentation in tolerogenic contexts. Furthermore, the inhibition of glycolysis upon TLR stimulation by 2-deoxyglucose, promotes Treg induction (93). Moreover, the ubiquitin-editing protein A20 is a crucial regulator of TLR-driven DC activation responsible for preventing autoimmunity (94). PRR-independent mechanisms of DC activation, such as through E-cadherin and β-catenin, lead to the induction of IL-10-producing T cells with autoimmune competency (95). Likewise, expression of the retinoic-acid synthesizing enzyme 2 (RALDH2) in DCs allows the synthesis and secretion of RA, a crucial regulator of Treg homeostasis (80, 81). Furthermore, additional physiological stimuli associated with the induction of tolerance, such as apoptotic bodies, have double functions: (i) their internalization by DCs induces tolerance itself; and (ii) these are a broad source of self-antigens that can be loaded onto MHC-II molecules and presented to CD4^+^ T cells (77). Additionally, specifically selected malignant cells produce IL-10, which is considered a mechanism of immune evasion since this cytokine inhibits MHC-I expression on cancer cells and strongly induces a tolerogenic phenotype on DCs by inducing IL-10 expression in a paracrine manner (96). Finally, pharmacological stimuli are able to trigger different transcription factors (e.g., GILZ, mTOR) that activate tolerogenic-associated protein expression on DCs (97, 98).

On the other hand, evidence indicates that signaling via PRRs in DCs also contributes to the establishment of lymphocyte tolerance via Tregs (Figure 1). Toll-like receptor 2 (TLR2) expression in DCs couples innate immunity to the induction of Foxp3^+^ T cells through activation of IL-10 and the RA-metabolizing enzyme retinaldehyde dehydrogenase 2 (91). Importantly, TLR2 signaling in DCs mediates suppression against autoimmunity via the induction of Tregs (91). In addition to TLR2, additional PRRs can induce IL-10 in DCs, also leading to the induction of Treg responses. The modulation of IL-10 secretion by DCs via the DC-SIGN receptor has been associated with Foxp3^+^ expression in naïve T cells in a murine model of nephrotoxic nephritis (92). Moreover, a monoclonal antibody to P-selectin, which also interacts with DC-SIGN, induces IL-10 secretion in human DCs that, in turn, contribute to the induction of CD4^+^CD25^+^Foxp3^+^ T cells (92).

Regulators of PRR signaling in DCs can also dictate the balance between T cell tolerance and immunity. The ubiquitin-editing enzyme A20, an anti-inflammatory protein involved in the attenuation of NF-κB signaling downstream of TLRs, is a crucial regulator of immune tolerance (Figure 1) (94). DC-specific deletion of A20 results in autoimmunity and systemic inflammation in mice, demonstrating the role of this protein as a key regulator in DC tolerance, which may be important to consider for clinical applications.

On the other hand, DCs able to induce Tregs can also be generated in the absence of PRR engagement. For instance, the maturation of DCs in response to the disruption of cell-to-cell interactions through the blockade of E-cadherin results in the induction of Tregs (95). This form of DC activation seems partially dependent on β-catenin (99). Interestingly, β-catenin signaling in DCs may also be a key controller of tolerance (83). The expression of β-catenin in DCs regulates the expression of RA, TGF-β, and IL-10 (Figure 1) (83). Importantly, selective ablation of β-catenin in CD11c-expressing cells leads to reduced Foxp3^+^ Tregs in the intestine (83), highlighting the contribution of specific signaling pathways in programing TolDCs. In this context, there are ongoing efforts aiming to identify gene expression signatures that distinguish between PRR-activated DCs and mature homeostatic DCs. These studies may shed light on the regulatory gene networks relevant to TolDC programing (5).

Besides changes in gene expression, the process of DC activation also involves a metabolic reprograming required to elicit T cell immunity. This process includes a rapid increase in glycolysis, which is essential to fulfill the biosynthetic and bioenergetic demands of activated DCs (93, 100). Inhibition of glycolysis in activated DCs through 2-deoxyglucose, an inhibitor of hexokinase, biases DCs to induce Foxp3^+^ T cells (93). This observation suggests that gaining better insights into the metabolic programs of steady-state versus activated DCs may be useful toward ultimately programing TolDCs for therapeutic use. In humans, TolDCs are characterized by increased catabolic pathway signaling, oxidative phosphorylation, mitochondrial oxidative activity, and fatty acid oxidation, as compared to activated DCs. In fact, the inhibition of fatty acid oxidation prevents the functioning of TolDCs and partially restores T cell stimulatory capacity (101). These data suggest that interfering with metabolic checkpoints in DC maturation may be an interesting strategy for programing TolDCs for therapeutic purposes.

## Pharmacological Modulation of TolDCs

The phenotypic plasticity of DCs can be modulated by a variety of stimuli with different nature and origin (Figure 1). Although there is currently no absolute consensus about the phenotype and characteristics of TolDCs, several studies support the notion that these cells must be able to induce a Foxp3^+^ Treg phenotype from naïve T cells (83, 84, 102, 103). From a clinical perspective, DC activation can be pharmacologically altered to obtain DCs capable of mediating T cell tolerance. Several cytokines, anti-inflammatory, and immunosuppressive drugs trigger a tolerogenic phenotype by interfering with checkpoints of DC differentiation and activation. These include glucocorticoids, vitamin D, aspirin, rapamycin, rosiglitazone, heme oxygenase-1 inducers, and andrographolide, among others (104–112). In addition, the ability of different cytokines to generate TolDCs has been tested, with IL-10 and TGF-β found as the most effective ­factors. DCs cultured in the presence of IL-10 and or TGF-β trigger anergy in naïve and memory T cells and are also able to activate T cells with an IL-2^low^, IFN-γ^low^, and IL-10^high^ Foxp3^+^ profile (10, 113). In fact, IL-10 is also involved in the differentiation of Foxp3^+^ Tregs in human *in vitro* models (11).

Regarding the pharmacological modulation of DCs in human, different protocols have been developed by adapting strategies used in mouse cells. Dexamethasone, vitamin D3, and rapamycin, in conjunction with the GM-CSF and IL-4, are the most used reagents in the *ex vivo* differentiation of peripheral blood monocytes into TolDCs (114). Dexamethasone, a glucocorticoid widely used to treat autoimmune diseases and graft rejection, mantains an immature DC phenotype that is associated with low expression of human leukocyte antigen II and co-stimulatory molecules. In addition, dexamethasone-treated DCs maintain expression of the chemokine receptor CCR7, suggesting that these DCs may retain migratory skills, a characteristic necessary for use in immunotherapy (115–117). Interestingly, dexamethasone stimulation in DCs also upregulates the glucocorticoid-induced leucine zipper (GILZ) protein and promotes the differentiation of antigen-specific CD25^(high)^Foxp3^+^CTLA-4/CD152^+^ and IL-10-producing Tregs able to inhibit CD4^+^ and CD8^+^ T cell responses (118). In fact, GILZ expression by DCs is an important regulator of Tregs (97). CD11c-GILZ^hi^ transgenic mice, which overexpress GILZ in the CD11c compartment, display an accumulation of Foxp3^+^ Tregs in spleen and in central and peripheral lymphoid organs of aged animals (97).

For its part, vitamin D3 (1,25-dihydroxyvitamin D_3_) promotes a tolerogenic phenotype in human monocyte-derived DCs via the activation of glucose metabolism. This effect is mediated by the PI3K/Akt/mTOR pathway, which controls the induction and maintenance of tolerogenic functions in DCs, stimulating the generation of Tregs (98, 119). In turn, rapamycin, an antibiotic of the macrolide family that possesses immunosuppressive properties, inhibits the maturation and effector functions of DCs. Indeed, murine rapamycin-treated DCs are inferior stimulators of syngeneic T cells that do not alter antigen uptake or *in vivo* homing to lymphoid tissue (111). Additionally, rapamycin treatment inhibits the ability of DCs to produce IL-12 and TNFα and can induce the proliferation of CD4^+^CD25^+^Foxp3^+^ Tregs (108, 120).

Taken together, the current evidence strongly supports that TolDCs generated by a single or combination of drugs could be an effective therapeutic approach to induce antigen specific Tregs in a variety of scenarios. In this regard, IL-10 and rapamycin appear to be the most suitable agents for generating TolDCs that could be applied in the treatment of human diseases.

## DC-Based Immunotherapy and the Induction of Tolerance: Where are We?

Over the last decade, different DC-based immunotherapies have been used to stimulate immune responses, and some have shown objective clinical benefits in patients with different types of cancer (121–125). Currently, several immunotherapeutic approaches are being studied, including for autoimmune conditions, allergies, and transplant rejection. All of these pathologies require permanent induction of T cell tolerance, as triggered by TolDC and orchestrated by proficient Treg functions. Indeed, the main target of TolDC immunotherapy should be the induction of tissue/organ infiltrating Tregs. Clinical data on TolDC-based immunotherapies remain limited, and the majority of studies are in preclinical stages. These studies focus on the development of safe and well-tolerated TolDCs with an anti-inflammatory phenotype and a preferential capacity to induce Tregs (126–128).

Regarding allergy treatments, atopic diseases are largely dominated by T helper type 2 immune responses that lead to an accumulation of eosinophils, immunoglobulin E production, and the sensitization of tissue mast cells (129). In *ex vivo* models, TolDCs… have shown to suppress the T helper type 2 immune response by inducing/expanding IL-10-expressing CD25^+^Foxp3^+^LAG-3^+^CTLA-4^+^ Tregs (130). Moreover, *in vitro* dexamethasone-induced human TolDCs are able to generate IL-10-expressing T cells specific for the Hev b5 antigen, a principal latex allergen (128).

On the other hand, autoimmune diseases are an issue of public health due to their heterogeneity and diverse clinical manifestations. Several studies in murine models of rheumatoid arthritis have demonstrated the efficacy of TNF-, IL-10-, and dexamethasone-treated TolDCs to treat collagen-induced arthritis, an experimental model for inflammatory joint diseases (131–133). In fact, the first prospective phase I clinical trial in patients with rheumatoid arthritis was recently concluded (134). In this trial, patients treated with autologous modified TolDCs and pulsed with citrullinated peptide antigens showed changes in the Treg/T effector ratio, where in 11 out of 15 treated patients, there was at least a 25% reduction in CD4^+^CD25^+^CD127^+^ T effector cells. However, Tregs increased by ≥25% in only 5 out of 15 treated patients (134). Importantly, this clinical trial has demonstrated the safety of this therapeutic approach as well as its effects on patients’ immunological parameters.

## Conclusion

In conclusion, and despite the substantial advances made in recent years regarding the development and function of TolDCs, it remains to be determined whether this knowledge can be applicable to the clinic. The ultimate success of TolDC-based therapies will be dictated by gaining in-depth understanding of the mechanisms by which TolDCs suppress autoreactive lymphocytes and induce Tregs; and by obtaining better insights regarding the mechanisms by TolDC suppress autoimmunity, allergies, and transplant rejection. These aspects include the stability/commitment of generated TolDC, especially in an *in vivo* inflammatory milieu, among others.

In addition, as there is no current consensus about which stimuli would be more reliable in inducing a committed TolDC phenotype, it is highly plausible that specific combination of these triggers, or even an additional stimuli yet to be identified, are needed to reach this phenotype. A third aspect to be addressed is the clear identification of specific receptors and the downstream signaling pathways that can be triggered in DCs to induce Treg responses. In this context, studies using high-throughput approaches (e.g., proteomics and genomics) will be of great help to identify the crucial genes/proteins networks involved in programing TolDCs suitable for Treg induction. This knowledge will assuredly facilitate the near-future design of reliable and clinically effective TolDCs for the treatment of several human diseases and conditions.

## Conflict of Interest Statement

The authors declare that the research was conducted in the absence of any commercial or financial relationships that could be construed as a potential conflict of interest.

## Abbreviations

APCs, antigen-presenting cells; cDCs, conventional dendritic cells; DCs, dendritic cells; GILZ, glucocorticoid-induced leucine zipper; iDCs, inflammatory dendritic cells; IL, interleukin; iTregs, inducible regulatory T cells; MHC, major histocompatibility complex; pDCs, plasmacytoid dendritic cells; PD-L1, programed cell death ligand-1; PRRs, pattern recognition receptors; RA, retinoic acid; TGF-β, transforming growth factor beta; TLR, toll-like receptor; TolDCs, tolerogenic dendritic cells; Tregs, regulatory T cells.
